# Influence of Electromagnetic Inductive Microcapsules on Self-Healing Ability of Limestone Calcined Clay Cement (LC3) Mortar

**DOI:** 10.3390/polym15143081

**Published:** 2023-07-18

**Authors:** Wei Du, Bo Liu, Zhengang Feng, Quantao Liu, Mingli Wu, Danying Zuo

**Affiliations:** 1 Hubei Engineering Research Center of Industrial Detonator Intelligent Assembly, Wuhan Textile University, Wuhan 430073, China; duwei@wtu.edu.cn (W.D.); zdy@wtu.edu.cn (D.Z.); 2 School of Materials Science and Engineering, Wuhan Textile University, Wuhan 430200, China; liubo13006311228@163.com; 3 Key Laboratory of Transport Industry of Road Structure and Materials, Chang’an University, Xi’an 710064, China; 4 State Key Laboratory of Silicate Materials for Architectures, Wuhan University of Technology, Wuhan 430070, China; 5 College of Life Science and Technology, Huazhong University of Science and Technology, Wuhan 430074, China; d202280823@hust.edu.cn

**Keywords:** self-healing, electromagnetic inductive, microcapsules, cracks, limestone calcined clay cement

## Abstract

In order to promote the sustainability of cementitious materials, it is imperative to reduce the level of environmental pollution and energy consumption during their production, as well as extend the service life of building elements. This study utilized limestone, calcined clay and gypsum as supplementary cementitious materials to prepare LC3 mortar, replacing 50% of ordinary silicate cement. Three types of microcapsules (M1, M2 and M3) were prepared using IPDI as a healing agent and polyethylene wax, polyethylene wax/nano-CaCO_3_ or polyethylene wax/ferrous powder as shell materials. The microcapsules were added to the LC3 mortar and tested for their effects on the mechanical properties, pore structure and permeability of mortars. Pre-loaded and pre-cracked mortar specimens were subjected to room temperature or under an applied magnetic field to evaluate the self-healing ability of the microcapsules on mortars. The kinetics of the curing reaction between IPDI and moisture were investigated using quasi-first-order and quasi-second-order reaction kinetic models. The experimental results showed that the mortar (S3) mixed with electromagnetic inductive microcapsules (M3) exhibited the best self-healing ability. The compressive strength retention, the percentage of pores larger than 0.1 μm, recovery of chloride diffusion coefficient and maximum amplitude after self-healing of S3 were 92.2%, 42.6%, 78.9% and 28.87 mV, respectively. Surface cracks with an initial width of 0.3~0.5 mm were healed within 24 h. The curing reaction between IPDI and moisture during self-healing followed a quasi-second-order reaction kinetic model.

## 1. Introduction

With the development of the global economy, there has been a substantial increase in the demand for infrastructure enhancement across various nations and regions. Consequently, this surge in demand has led to a corresponding rise in the consumption of cement [1]. However, the cement industry itself is known for its polluting and carbon-intensive manufacturing processes, making it a significant contributor to carbon emissions in countries worldwide [2]. In light of the growing emphasis on environmental protection, countries around the globe are increasingly advocating for stronger environmental regulations within the cement industry. Many nations have already initiated efforts to promote the adoption of low-carbon technologies, aiming to reduce carbon emissions and energy consumption in order to meet stringent environmental requirements [3,4].

The incorporation of calcined clay and limestone as supplementary cementitious materials (SCMs) in composite blends offers a promising solution to reduce carbon emissions (up to 30%) and minimize the usage of silicate cement clinker in the cement manufacturing process [5,6]. This approach aligns with the contemporary ideals of green ecology and sustainable development [7]. The concept of limestone calcined clay cement (LC3) was introduced by Professor Scrivener at the Swiss Federal Institute of Technology in Lausanne [8]. LC3 is a silicate-based cement that relies on limestone and calcined clay, utilizing abundant reserves of low-grade clay. Experimental investigations have demonstrated that LC3 enhances the performance of cementitious materials through the reaction with volcanic ash and the interaction between limestone and clay [9,10]. Notably, LC3 can be seamlessly integrated into existing construction methods without requiring significant modifications. The inclusion of calcined clay and limestone in cementitious materials leads to substantial optimization of the pore structure, resulting in reduced porosity. As a result, the diffusion and intrusion of harmful substances are effectively impeded, enhancing the durability of cement-based materials against chloride ion attacks. Previous research [11,12] has demonstrated that the chloride diffusion coefficient of composite cementitious systems containing calcined clay and limestone is lower than that of conventional silicate cement under equivalent conditions. Consequently, this innovative low-carbon cement system exhibits significant potential for widespread application [13].

Despite the advantages offered by cementitious materials, including LC3, the occurrence of cracks over time remains a concern due to various external factors such as ionic attack, loading and freeze-thaw cycles [14,15,16]. Cracks not only diminish the strength of cement-based materials but also pose a risk to the structural integrity of buildings, thereby compromising safety [17,18]. Presently, most solutions for addressing these damage issues involve manual repairs, which are costly and labor-intensive [19]. Moreover, in underground structures, hydroelectric or nuclear power plants, repairing damages can be particularly challenging. Consequently, there is an urgent need for intelligent cement-based materials capable of autonomously detecting and repairing damages, known as self-healing cement-based materials [20]. Since the publication of White’s study in 2001 [21], microcapsule technology has been widely employed for the self-healing of concrete. Microcapsules possess a core-shell structure, wherein healing agents and curing agents are encapsulated. When cracks form in cement-based materials, the microcapsules rupture at the crack site. The healing agent infiltrates the crack and reacts with the curing agent, resulting in the formation of a highly adhesive solid that seals the crack and prevents further propagation [22]. Currently, there are two types of mechanisms for microcapsule rupture: active and passive. An active fracture occurs when microcapsules rupture due to stress at the crack tip within the matrix, leading to the release of healing agents. However, a challenge lies in the fact that microcapsules can only break under stress at the crack tip, limiting their effectiveness in repairing cracks. Passive microcapsule rupture has recently gained significant attention. This refers to the rupture of microcapsules triggered by external stimuli, allowing for better utilization of the self-healing ability of microcapsules [23,24].

The use of SCMs to replace a portion of the cement clinker in the production of LC3, and subsequently mixing microcapsules to prepare a novel type of low-carbon cement-based material with crack self-healing capabilities, offers several advantages:

1. Reduced carbon footprint: The incorporation of SCMs in cement production reduces the amount of cement clinker required, thereby reducing the carbon footprint of the material [25].
2. Improved durability: The addition of microcapsules enhances the durability of the material by enabling it to self-heal cracks, thereby increasing its lifespan.
3. Cost-effective: The use of SCMs is cost-effective as they are readily available and can be obtained at a lower cost than cement clinker.
4. Sustainable: The use of SCMs and microcapsules promotes sustainability by reducing the environmental impact of cement production and extending the lifespan of the resulting material.

Overall, the use of SCMs and microcapsules in the production of low-carbon cement-based materials with crack self-healing capabilities is a promising approach for enhancing the sustainability and durability of construction materials.

Several studies have investigated the use of LC3 cement-based materials and microencapsulated self-healing technologies, demonstrating promising outcomes [26,27,28,29]. However, there remains a research gap concerning LC3 self-healing cement-based materials, which hampers their widespread implementation. In practical applications, the stress at the crack tip is often insufficient to rupture the microcapsules, thereby limiting their self-healing effectiveness. Introducing an external magnetic field can overcome this limitation by inducing electromagnetic heating, which melts the microcapsules and releases the healing agents. This allows for the controllable and rapid repair of concrete cracks using microcapsules. To address this issue, this study focuses on the development of electromagnetic induction microcapsules. The healing agent chosen is IPDI, while the shell material consists of polyethylene wax and ferrous powder. These microcapsules are separately incorporated into LC3 mortar, and their impact on the mechanical properties, pore structure and permeability of the mortar is evaluated. Additionally, some mortars are pre-loaded and pre-cracked, and then subjected to room temperature or an applied magnetic field to assess the self-healing ability of the microcapsules. The kinetics of the curing reaction between IPDI and moisture during the self-healing process are analyzed using quasi-first-order and quasi-second-order reaction kinetic models. This analysis aims to investigate the rate of the curing reaction during the self-healing process.

## 2. Materials and Methods 

### 2.1. Materials

Polyethylene wax (melting temperature 100–115 °C), nano-CaCO_3_ (particle size 50 nm) and ferrous powder (particle size 50 nm) were bought from Nanjing Tinashe New Material Technologies Co., Ltd., Nanjing, China; Isophorone diisocyanate (IPDI) and perfluoro-tributylamine were acquired from Guangzhou Haye New Material Technology Co., Ltd., Guangzhou, China. Ordinary silicate cement 42.5R was supplied by China Resources Cement Co., Ltd., Hong Kong. Clay was calcined at 800 °C using a previously published method [30], with a kaolinite content of 27%. The amount of clay was calcined in one batch. Finally, the calcined clay was ground with a ball mill. Limestone powder was purchased from the Wuhan Haitong lime factory. Gypsum was applied by Yancheng Hucheng Gypsum Products Co., Ltd., Guangzhou, China. Table 1 shows a summary of the chemical composition. River sand (modulus: 2.35) was provided by Jiangxi Kangkong Environmental Protection Materials Co., Ltd., Yi Chun, China. 

The chemical composition of the powders was analyzed with X-ray fluorescence (XRF, ZSX Primus II). The XRF fused cast bead method (ISO 12677:2003) was used in the sample preparation system (XRF-Scientific, Montreal, Canada). Beads were prepared by mixing ∼4 g each of lithium tetraborate and lithium metaborate with a test sample (∼0.8 g). The resistance furnace was heated to a fixed temperature of (1025 ± 25) °C.

**Figure 1:**
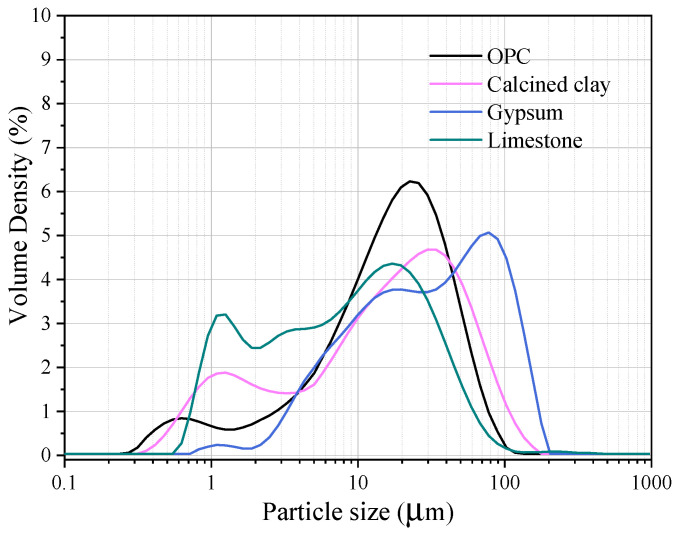
Particle size distributions of OPC, calcined, gypsum and limestone.

Through particle size distribution (PSD) analysis (Figure 1), the average particle sizes of OPC, calcined, gypsum and limestone were measured at 18.5, 12.2, 13.6 and 15.8 µm, respectively. Their average particle sizes are essentially identical.

### 2.2. Fabrication of Microcapsules 

**Microcapsule 1 (M1):**

A three-neck flask was used to melt 40 g of polyethylene wax in an oil bath at 125 °C. Subsequently, 60 g of IPDI was added to the flask, and the resulting mixture was agitated for 150 min at 900 rpm. To induce a sharp drop in temperature, 400 mL of perfluoro-tributylamine was added to the flask, resulting in the formation of microcapsules (polyethylene wax-coated IPDI). The microcapsule suspension was then subjected to 40 min of ultrasound oscillation before being filtered.

**Microcapsule 2 (M2):**

The fabrication method used was identical to M1. Specifically, 35 g of polyethylene wax and 5 g of nano-CaCO_3_ were utilized to form the microcapsule shell.

**Microcapsule 3 (M3):**

The fabrication method used was identical to M1. Specifically, a microcapsule shell was formed using 35 g of polyethylene wax and 5 g of ferrous powder.

Table 2 presents the various indicators of microcapsules. 

### 2.3. Mortar Preparation 

Table 3 specifies the water-cement and sand-cement ratios for the mortar. To prepare the mortar, the mixing pot was loaded with cement, sand and microcapsules, which were mixed at 90 rpm for 90 s to form a homogeneous mixture. The mixing pot was then filled with water, and the resulting homogeneous mixture was agitated at 150 rpm for 90 s, resulting in the production of a fresh mortar. Fresh mortar was poured into molds coated with a release agent, with dimensions of 70.7 mm × 70.7 mm × 70.7 mm and Φ 100 mm × 200 mm, respectively. Then, 24 h later, the molds were released and the specimens were cured at 95 ± 5% RH and 20 ± 2 °C for 28 days.

### 2.4. Self-Healing of Mortars (Mechanical Properties)

**Figure 2:**
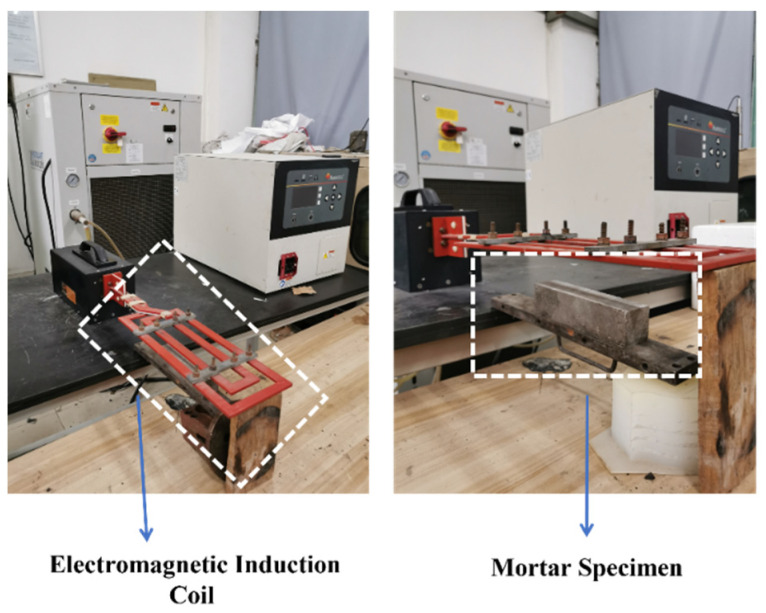
Electromagnetic induction heating system.

**Figure 3:**
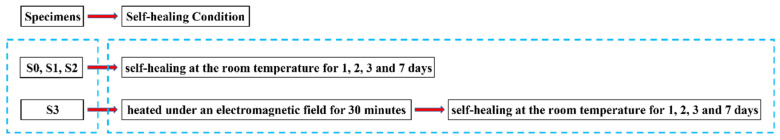
Self-healing condition of S0–S3.

The specimens, after 28 days of standard curing, were taken out. The compressive strengths of each group were initially tested and denoted as f_0_. Subsequently, each group was pre-loaded with 80% of f_0_. Then, specimens S0, S1 and S2 were kept at room temperature for 1, 2, 3 and 7 days. Specimen S3 was subjected to an electromagnetic field for 30 min (Figure 2) with an output voltage of 600 V and a field frequency of 124 kHz. The heating coil was positioned 0.5 cm away from the upper surface of S3. After heating, S3 was left at room temperature for 1, 2, 3 and 7 days. The self-healing condition is depicted in Figure 3. Finally, the mortar was reloaded and the compressive strength retention was calculated using Equation (1).

(1) $$\varepsilon =\frac{f_{e}}{f_{0}}\times 100\%$$

where ε is the compressive strength retention of the mortar, f_0_ is the initial compressive strength of the mortar and f_e_ is the compressive strength of the mortar after self-healing.

### 2.5. Curing Reaction Kinetics

To describe the specific curing reaction processes of IDPI and moisture during self-healing, we utilized a quasi-first-order reaction kinetic model and a quasi-second-order reaction kinetic model. These models were utilized to simulate the quasi-first-order kinetic rate in Equation (2) and the quasi-second-order reaction kinetic rate in Equation (3).

(2) $${\ln(p}_{0}{-p}_{t}{)=\mathrm{lnp}}_{0}{-K}_{1}t$$

(3) $$\frac{t}{p_{t}}=\frac{1}{K_{2}p_{0}^{2}}+\frac{t}{p_{0}}$$

where K_1_ is the quasi-first-order reaction rate constant (d^−1^), K_2_ is the quasi-second-order reaction rate constant (d^−2^), p_0_ is the initial compressive strength retention (%), p_t_ is the compressive strength retention (%) at t time.

### 2.6. Nuclear Magnetic Resonance Test

The MicroMR23-025V NMR instrument was utilized to test the pore size distributions of mortars. The NMR technique is capable of testing the pore size distributions of mortars due to the molecular resonance phenomenon of water in cement within the magnetic field. The exchange and release of energy result in relaxation behavior, and the length of this relaxation time can indicate the size of the pores. After an 80% f_0_ pre-load and a self-healing period of 3 days, the pore size distributions of S0–S3 were measured. Please refer to the supplementary material for the specific testing process.

### 2.7. Permeability Test

The chloride diffusion coefficient of specimens was tested following the standard NT Build 443 [31]. Each group of specimens (S0–S3) was subjected to 80% f_0_ loading, and these specimens were allowed to self-heal for 1, 2, 3 and 7 days. The recovery was calculated using Equation (4).

(4) $$\sigma =-\frac{\sigma_{e}-\sigma_{0}}{\sigma_{0}}\times 100\%$$

where *σ* is the chloride diffusion coefficient recovery rate (%), *σ_0_* is the chloride diffusion coefficient of mortars after pre-loading (10^−12^ m^2^/s) and *σ_e_* is the chloride diffusion coefficient of the pre-loaded mortars after self-healing (10^−12^ m^2^/s). 

### 2.8. Ultrasonic Testing

Cement-based materials are heterogeneous composite materials with an inhomogeneous texture, a large number of pores and a complex structure. The structures made of cement-based materials used in various engineering projects are generally large in volume and complex in structure. Therefore, non-destructive testing (NDT) techniques are gradually gaining attention worldwide. NDT does not damage cement-based materials’ structure components and can obtain the most needed information regarding the physical quantity of cement-based materials. The test operation is simple, not limited by the shape and size of the structure and can be repeated several times. It can also be used for important structural parts of long-term monitoring to reduce losses and avoid accidents. In this study, ultrasound waves are generated and transmitted through a generator and received using an oscilloscope (MDO 3024, Tektronix, China Ltd., Shanghai, China). Specific test procedures are described in the Supplementary Material.

### 2.9. Surface Crack Self-Healing 

**Figure 4:**
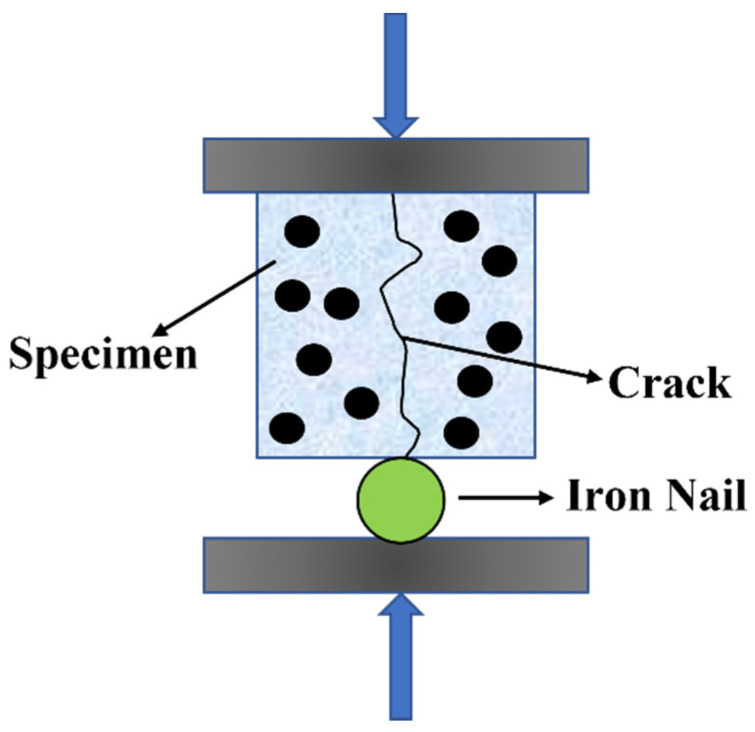
Schematic diagram of mortars pre-cracking test.

The pre-cracking of specimens was performed using automatic compression equipment at a constant rate of 10 N/s when they reached 28 days of age (refer to Figure 4). The variation in crack width from S0–S3 was tested according to the self-healing conditions shown in Figure 3. The pixel number included in the crack images of cracked specimens, i.e., the cracked area with different self-healing times, was calculated using image analysis software. The healing rate of the cracked area was then calculated using Equation (5).

(5) $$\eta_{\mathrm{crack}}=\frac{A_{0}-A_{t}}{A_{0}}\;\times \;100\%$$

where η_crack_ is the healing rate of the crack area (%); A_0_ is the number of initial crack pixels, A_t_ is the number of crack pixels after self-healing.

## 3. Results and Discussion 

### 3.1. Compressive Strength Retention

**Figure 5:**
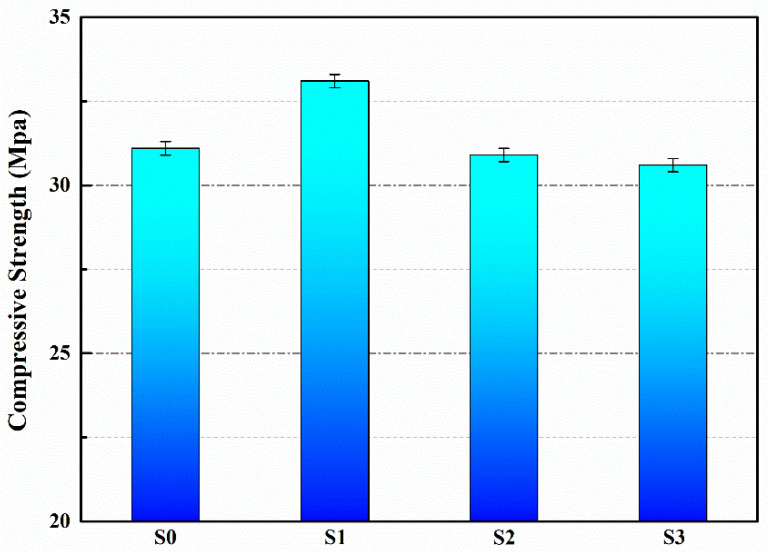
Compressive strengths of S0–S3.

The compressive strengths of the mortar specimens from each group after 28 days of curing are presented in Figure 5. The results reveal that specimens S0, S1, S2 and S3 exhibited compressive strengths of 31.1 MPa, 33.1 MPa, 30.9 MPa and 30.6 MPa, respectively. In comparison to S0, the compressive strength of S1 increased by 6.4%. However, there was only a slight, non-significant decrease in the compressive strengths of S2 and S3. The improvement in compressive strength observed in S1 can be attributed to the small particle size of M1 (microcapsules present in S1), which acted as fine aggregates and dispersed stresses upon incorporation into the mortar. Furthermore, the porous structure of the mortar facilitated the filling of some pores by M1, thereby enhancing the compactness of the mortar and subsequently increasing its compressive strength [32]. On the other hand, due to their larger particle sizes, M2 and M3 were not as effective in filling the micro-pores within the mortar compared to M1. As a result, there was almost no change in the compressive strength of S2 and S3 when compared to S0.

**Figure 6:**
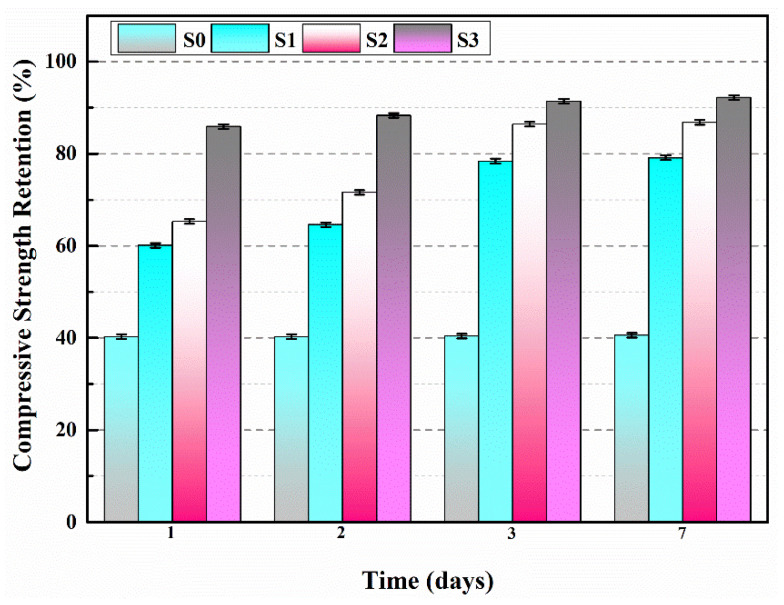
The compressive strength retention of S0–S3.

Figure 6 illustrates the retention of compressive strength for specimens S0–S3 at various curing periods following pre-damage. As illustrated in Figure 6, the compressive strength retention of specimens S1, S2 and S3 was consistently higher than that of the blank specimen, S0, throughout all stages of the self-healing process.

**Figure 7:**
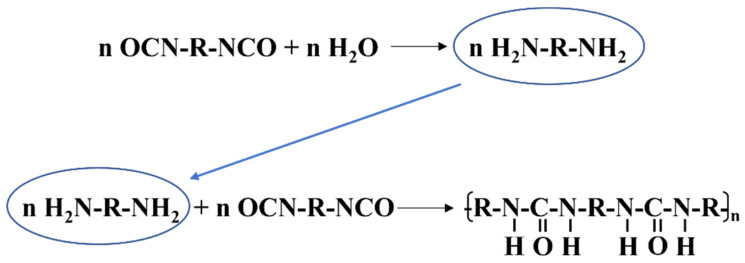
The chemical reaction process of diisocyanate with water.

During the 24-h period from day 2 to day 3 of the self-healing process, noticeable changes in the compressive strengths of S1 and S2 were observed. By day 3, the compressive strengths of S1 and S2 had recovered to 78.4% and 86.4%, respectively. This recovery can be attributed to the presence of microcracks in the mortar after pre-damage. The stress at the crack tips led to the rupture of microcapsules in close proximity to the cracks, releasing the encapsulated IPDI. Subsequently, the IPDI flowed into the cracks and rapidly reacted with the moisture present, resulting in the formation of polyurea (as shown in Figure 7). Polyurea effectively filled the cracks, thereby restoring the mechanical properties of the mortars. From Table 2, it can be observed that M2 has a higher core content compared to M1, ensuring a greater amount of effective IPDI available for participating in the self-healing reaction. Consequently, S2 exhibited a higher retention of compressive strength than S1.

Figure 6 illustrates that S3 exhibited a remarkable recovery of its mechanical properties after one day of self-healing, with a compressive strength retention of 85.9%. This surpasses the data of S1 and S2, which recorded compressive strength retentions of 60.1% and 65.3%, respectively. This result confirms that the application of an electromagnetic field promotes the self-healing of pre-damaged mortars. The external alternating magnetic field induces the generation of heat in the ferrous powder within the microcapsule shell material, primarily due to eddy current loss and hysteresis loss [33]. This heat both melts and ruptures the microcapsules, leading to the release of IPDI and accelerating the reaction rate between IPDI and moisture in the mortars. In essence, the heating from the applied magnetic field enhances the rupture rate of the microcapsules, facilitating a self-healing process with a higher reaction rate, thereby improving the overall self-healing efficiency. By day 3, the compressive strength retention of S3 had reached 92.2%, significantly surpassing that of S1 and S2 during the same period. This can be attributed to the unpredictable direction of crack propagation within the pre-damaged mortar. The self-healing capacity of the mortar may be compromised if microcapsules are absent during crack propagation or if the number of microcapsules is insufficient. In contrast, the IPDI within M3 could smoothly flow into microcracks at various locations within the mortar after the melting of the capsule shells. This occurred regardless of the initial distribution of the microcapsules, enabling accurate and rapid healing of the cracks and ultimately restoring the mechanical properties of the mortars. From day 3 to day 7, the compressive strength retentions of S1, S2 and S3 exhibited minimal changes, indicating that the mechanical properties of the mortars had essentially recovered after three days of self-healing.

Therefore, microcapsules can enhance the compressive strength retention of mortars, and the self-healing ability of internal damage of the mortar is in the order of S1 < S2 < S3. 

### 3.2. Curing Reaction Kinetics 

**Figure 8:**
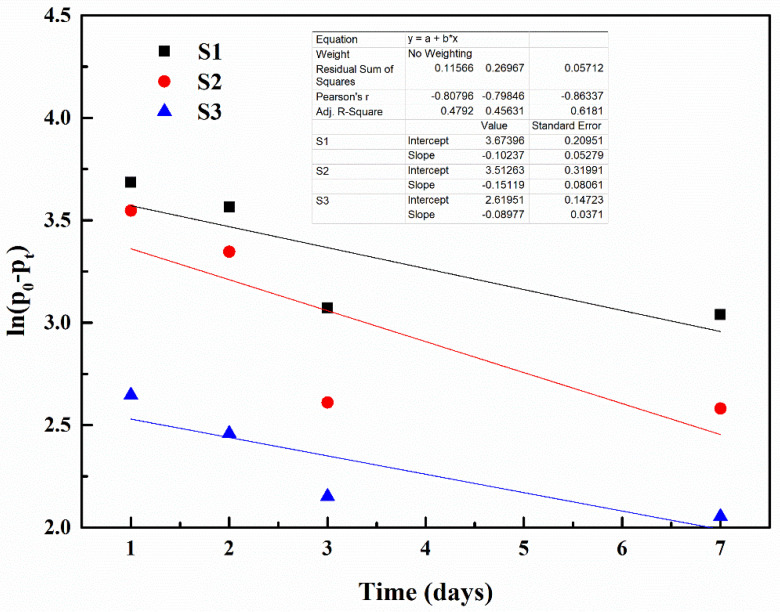
Quasi-first-order curing reaction kinetics.

**Figure 9:**
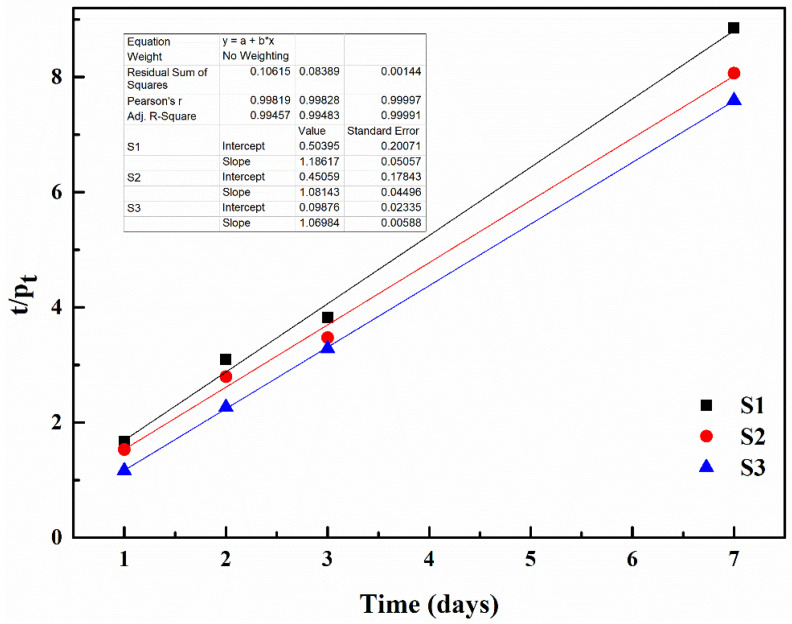
Quasi-second-order curing reaction kinetics.

Figure 8 and Figure 9 display the linear fit diagrams representing the quasi-first-order and quasi-second-order curing reaction kinetics, respectively, of the IPDI reaction with moisture during the self-healing process of the pre-damaged S1, S2 and S3 mortars, as per the conditions outlined in Figure 3. Table 4 provides the quasi-first-order reaction rate constants (K_1_) and quasi-second-order reaction rate constants (K_2_), calculated using Equations (2) and (3), respectively. The R^2^ values range from 0.479 to 0.618 after linear fitting based on the quasi-first-order reaction kinetics. In contrast, the R^2^ values exceed 0.995 after linear fitting using the quasi-second-order reaction kinetics. These results indicate that the quasi-second-order linear equation accurately describes the relationship between the curing reaction of IPDI and moisture. Therefore, it can be concluded that the curing reaction of IPDI with moisture in the self-healing process of pre-damaged mortar follows a quasi-second-order reaction.

Table 4 demonstrates that the K_2_ value for S3 (6.098) is significantly higher compared to S1 and S2 (1.506 and 1.883, respectively). Higher K_2_ values indicate faster curing reactions between IPDI and moisture, resulting in improved self-healing effects. This improvement can be attributed to the inclusion of M3, which contains ferrous powder on its surface, in S3. The ferrous powder possesses excellent magnetic permeability and generates heat when exposed to an external electromagnetic field. This heat causes the microcapsules to melt and rupture, releasing IPDI. Additionally, the temperature in the surrounding environment increases due to heat generation, leading to enhanced molecular movement and accelerated reaction rates between IPDI and moisture. Consequently, the formation of polyurea is expedited, thereby enhancing the self-healing capability of the microcapsules in the mortars. The investigation into self-healing kinetics reveals that pre-damaged mortar exhibits the most effective self-healing property when influenced by an external magnetic field.

### 3.3. Pore Size Distribution

Pores larger than 0.1 μm, which negatively impact the mechanical properties and durability of cementitious materials [34], are the focus of this section. The impact of microcapsules on these pores in mortars is discussed.

**Figure 10:**
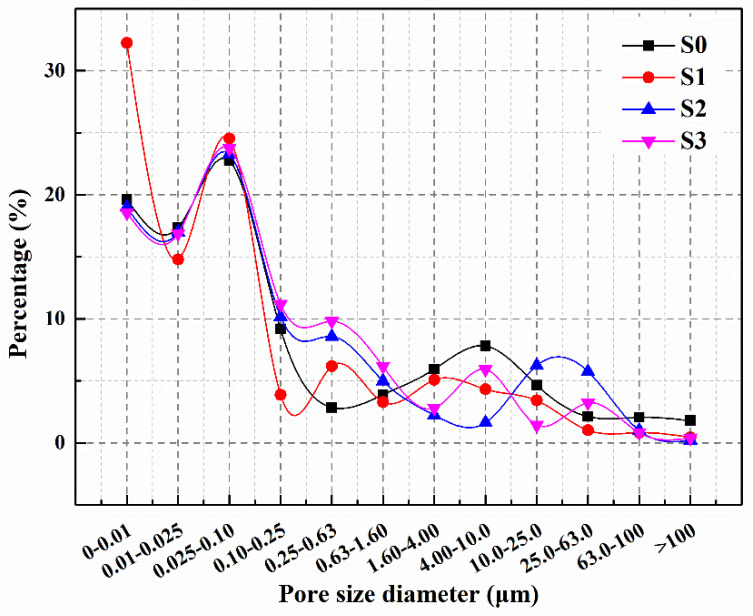
Pore size distribution of S0–S3.

Figure 10 illustrates that, at 28 days of age, the percentage of pores larger than 0.1 μm in S0 was 40.3%, while in S1 it was 30.4%. The percentage of macropores in S1 was reduced by 24.4% compared to S0, primarily because the relatively small particle size of M1 filled the micropores in the mortar, reducing internal defects and making the mortar structure denser. At 28 days of age, the percentages of pores larger than 0.1 μm were 40.8% and 41.8% for S2 and S3, respectively. Compared to S0, the percentage of macropores increased by 1.3% and 3.7% for S2 and S3, respectively. This increase is due to the larger particle size of M2 and M3, which affects the particle gradation of the mortar and slightly increases the percentage of macropores. However, the increase in macropores percentage in S2 and S3 is limited, and its effect on the internal structure of the mortar is not significant.

**Figure 11:**
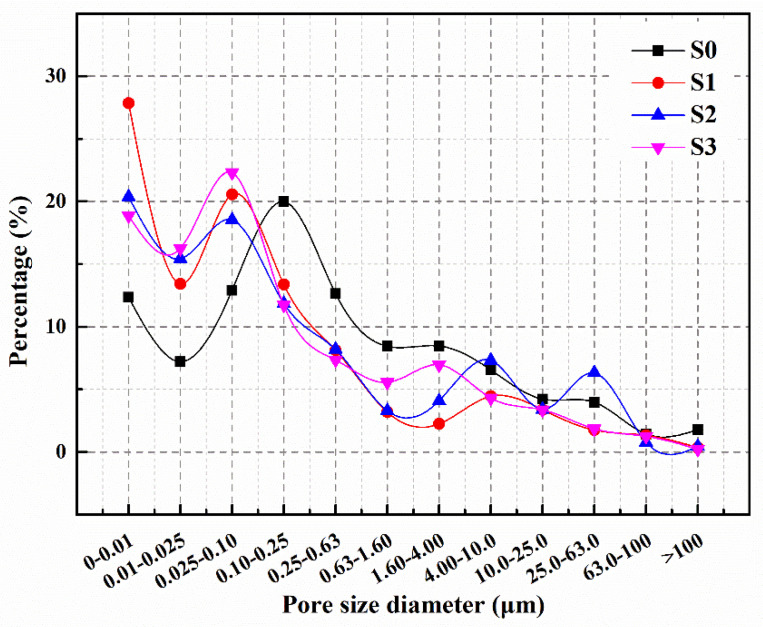
Pore size distribution of S0–S3 after self-healing.

Figure 11 illustrates the pore size distributions of pre-damaged mortars after three days of self-healing. The percentage of pores larger than 0.1 μm in S0 increased to 67.5%, indicating a significant increase compared to the initial value. This suggests that the blank specimen without microcapsules lacked the inherent self-healing ability to repair internal damage. In S1, S2 and S3, the percentage of macropores was 38.2%, 45.7% and 42.6%, respectively. These results indicate that M3 can greatly enhance the self-healing capacity of the mortar, as the proportion of macropores in S3 is more similar to the initial value compared to S1 and S2. This improvement is attributed to the damage to the shell material of the microcapsules caused by the stress at the tip of the microcrack, leading to the release of IPDI. The reaction between IPDI and moisture in the specimens rapidly generates polyurea, which heals the microcracks and reduces the percentage of macropores in the mortar. However, crack extension is unpredictable, and if microcapsules are not encountered along the crack path or are only encountered after significant cracking has occurred, the healing of cracks may be delayed, negatively impacting the self-healing effect. The shell material of M3 contains ferrous powder, which generates heat to melt and rupture the microcapsules under the applied magnetic field. With its low viscosity, IPDI can easily flow into the cracks and quickly react with moisture at higher temperatures, thereby improving self-healing efficiency in terms of timeliness and precision. This promotes the rapid healing of cracks inside the damaged mortar and reduces the percentage of macropores [35].

### 3.4. Recovery of Chloride Diffusion Coefficient

**Figure 12:**
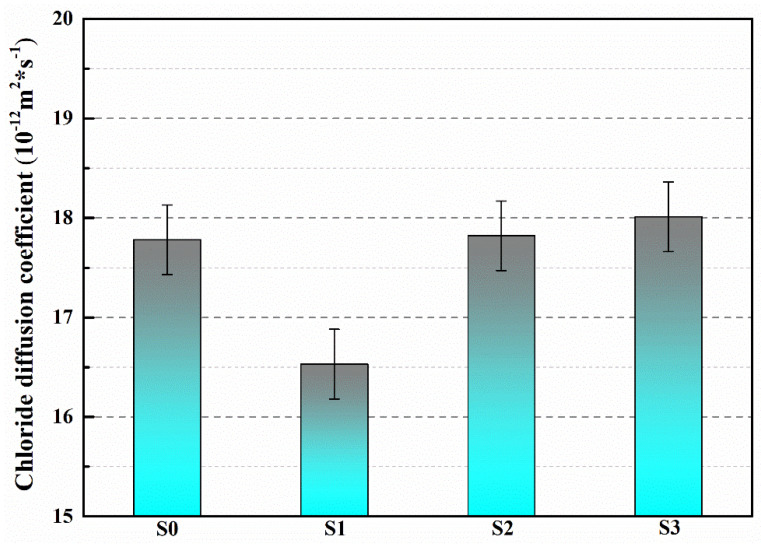
Chloride diffusion coefficient of S0–S3.

The chloride diffusion coefficients can be utilized to assess the compactness of mortar, reflecting the filling effect of microcapsules on microcracks and pores in cement-based materials. The chloride diffusion coefficients of S0, S1, S2 and S3 at 28 days of age are shown in Figure 12. The chloride diffusion coefficient of S0 is 17.78 × 10^−12^ m^2^/s, while that of S1 is 16.58 × 10^−12^ m^2^/s, indicating a 6.7% reduction compared to S0. This suggests that during the molding process, the microcapsules fill the pores and defects in mortar, making it dense and improving its impermeability. The chloride diffusion coefficients of S2 and S3 were 17.82 × 10^−12^ m^2^/s and 18.01 × 10^−12^ m^2^/s, respectively, which were slightly decreased compared to S0, but not significantly so. This is due to the relatively larger particle size of M2 and M3, which has a certain influence on the grading of the mortar. The filling effect is not as good as that of M1, which has a smaller particle size, providing a channel for chloride ion flow transmission and reducing the compactness of the mortar, leading to a decrease in its impermeability [36].

**Figure 13:**
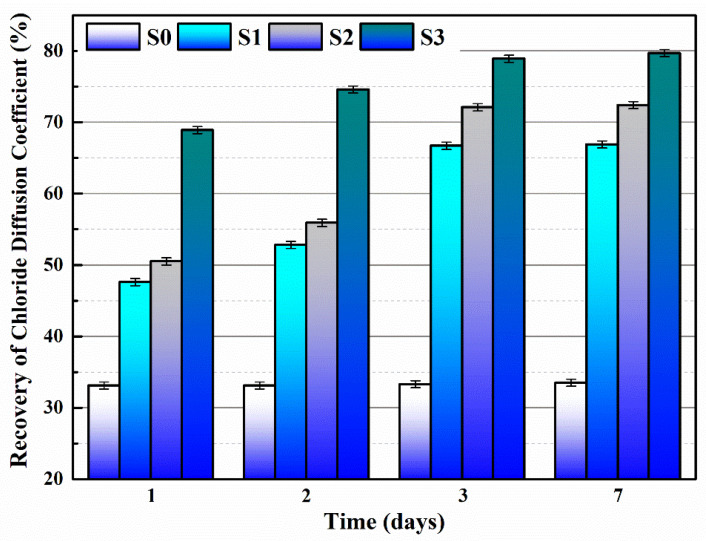
The recovery of the chloride diffusion coefficient of S0–S3 after self-healing.

Figure 13 illustrates the recovery of the chloride diffusion coefficients of mortars doped with microcapsules. When the pre-load was 80% f_0_, the chloride diffusion coefficient of S0 did not recover, even after 7 days of self-healing. This indicates that the cementitious material alone was insufficient to repair the damaged structure inside the mortar. The recovery of the chloride diffusion coefficient of S1 and S2 significantly improved within the 24-h time period from 2 to 3 days of self-healing, finally reaching 66.7% and 72.1%, respectively. This is because cracks appear inside the mortar after being subjected to external preload, but it takes some time for the cracks to extend until they encounter the microcapsules. M2 has a higher core content than M1, and the core leakage rate is lower after 60 days, making it more effective for mortar self-healing. Unlike S1 and S2, S3 can reach 68.9% recovery of its chloride diffusion coefficient after one day and 78.9% directly after three days. This is because, in addition to crack extensions triggering microcapsule rupture, the magnetic material (ferrous powder) in the M3 shell material generates heat under the action of the applied magnetic field, leading to the melting and rupture of microcapsules. The reaction between IPDI and moisture is more intense under high temperatures, which can quickly generate more polyurea, thus efficiently recovering the impermeability of the mortar [37]. The experimental results are consistent with the recovery effect of mortar compressive strength in Section 3.1.

### 3.5. Ultrasonic Testing

**Figure 14:**
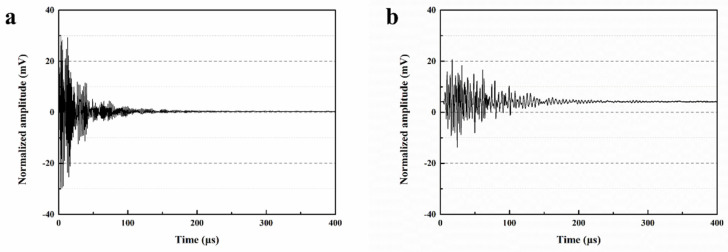

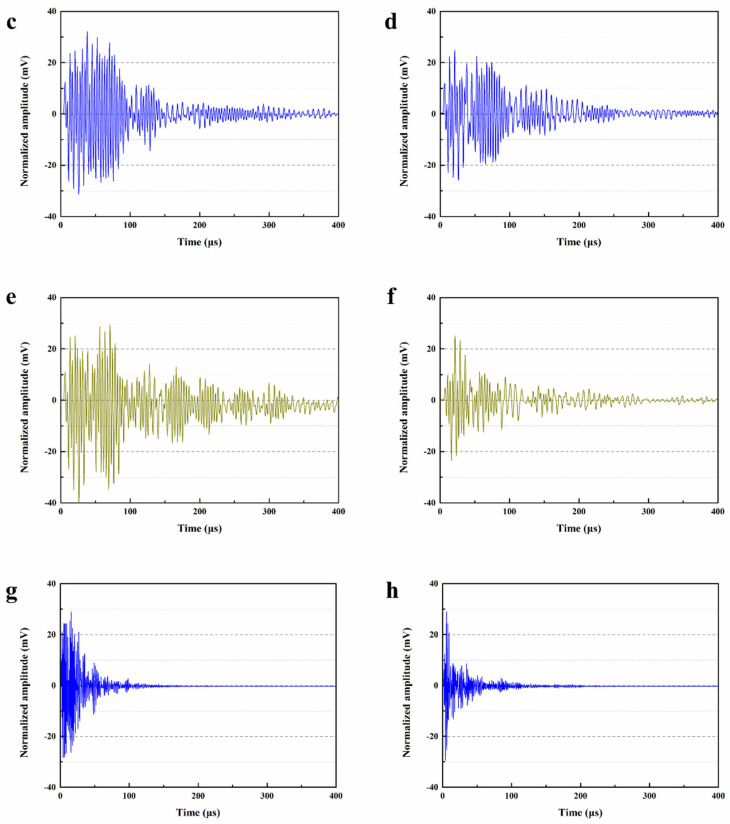
Ultrasonic waveform of S0–S3. (**a**) the initial ultrasonic waveform of S0, (**b**) the ultrasonic waveform of self-healed S0, (**c**) the initial ultrasonic waveform of S1, (**d**) the ultrasonic waveform of self-healed S1, (**e**) the initial ultrasonic waveform of S2, (**f**) the ultrasonic waveform of self-healed S2, (**g**) the initial ultrasonic waveform of S3, (**h**) the ultrasonic waveform of self-healed S3.

The waveform of transmitted ultrasound is influenced by the transmission medium [38]. This experiment assesses the self-healing ability of mortars by transmitting ultrasound and presents the initial and self-healed ultrasonic waveforms for S0, S1, S2 and S3 in Figure 14. The initial maximum amplitudes of S0, S1, S2 and S3 were 29.96 mV, 33.14 mV, 29.93 mV and 28.91 mV, respectively. The maximum amplitude of S1 increased by 10.6% compared to S0, while the maximum amplitudes of S2 and S3 decreased by 0.1% and 3.5%, respectively. When ultrasound propagates through mortar, it experiences significant acoustic impedance, which is much greater than in air. Therefore, when there are pores or defects in mortars, the ultrasonic signal undergoes attenuation, resulting in a reduced amplitude at the signal receiver [39]. The higher internal compactness of mortar reduces the attenuation of ultrasonic signals during propagation, leading to a larger wave amplitude at the receiver. As shown in Figure 10, the percentage of pores larger than 0.1 µm in S1 is lower than that in S0, indicating a denser internal structure, which results in a higher maximum amplitude for S1 compared to S0. Although the percentage of pores larger than 0.1 µm increases in S2 and S3, and the maximum amplitude tends to decrease, it has minimal effect on their mechanical and permeability properties (Section 3.1 and Section 3.4), and therefore, it does not significantly impact engineering applications.

After the self-healing process, the maximum amplitudes of ultrasonic signals for S0, S1, S2 and S3 were measured as 20.02 mV, 26.01 mV, 25.99 mV and 28.87 mV, respectively. However, the application of external pre-loads resulted in the formation of cracks inside the mortar, leading to the attenuation of ultrasonic signals and a significant reduction in the maximum amplitude of S0. These observations indicate that the microcapsules possess remarkable restorative capabilities for the internal structure of the mortar, as demonstrated by the substantial recovery of maximum amplitudes in S1, S2 and S3 following self-healing. Notably, the maximum amplitude of S3 nearly reached its initial value. This can be attributed to the encounter of crack propagation with the microcapsules, causing their rupture and the subsequent release of the healing agent. Compared to M1, M2 has a higher core content, resulting in S2 (containing M2) exhibiting a stronger self-healing ability than S1. Moreover, M3, which includes ferrous powder in the capsule shell, generates heat under the applied magnetic field, facilitating the melting of microcapsules and elevating the reaction temperature between IPDI and moisture. This process leads to the production of more polyurea, thus enhancing the self-healing efficiency of microcapsules in mortars. As a result, internal defects such as cracks are reduced in S3, leading to an increased maximum amplitude.

### 3.6. Crack Self-Healing

#### 3.6.1. Visualization of Crack Filling

**Figure 15:**
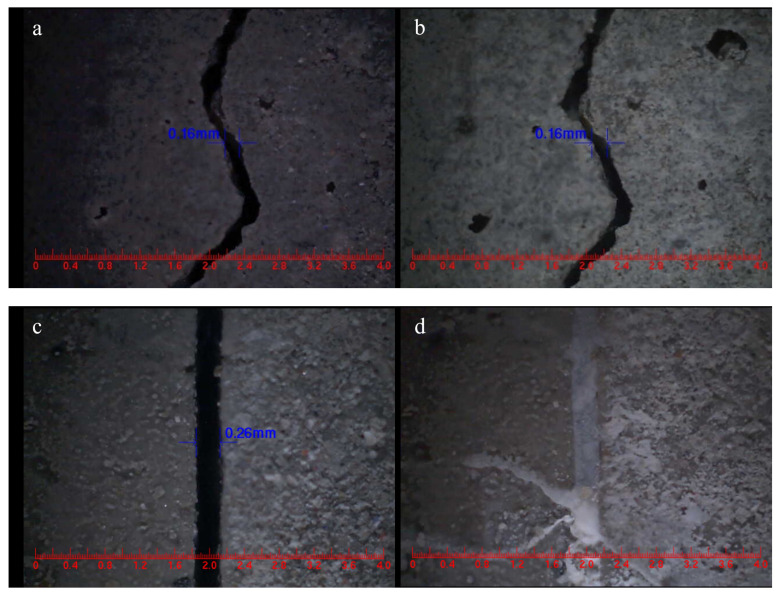

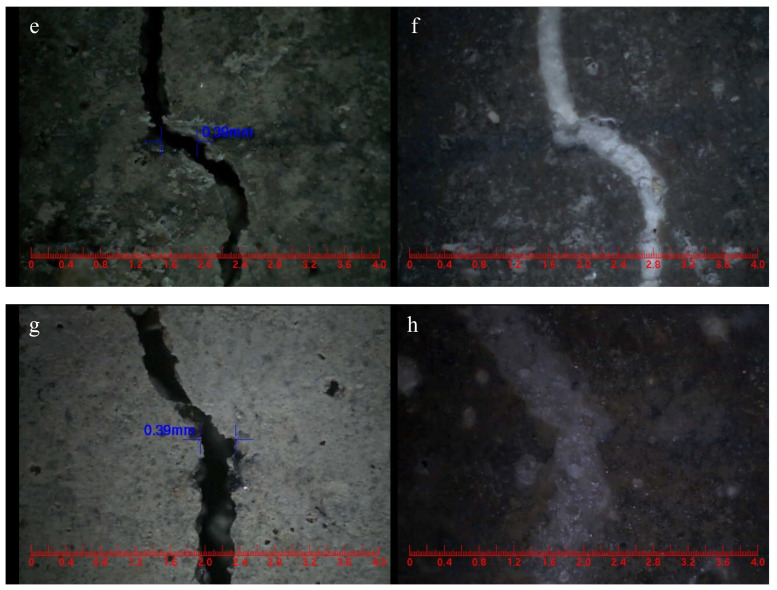
Self-healing of surface cracks.

The surfaces of S0, S1, S2 and S3 were intentionally prepared with cracks using the splitting method. After undergoing self-healing under different conditions for 48 h, the changes in crack widths were measured and are presented in Figure 15. From the observations in Figure 15a,b, it is evident that cracks with an initial width of 0.16 mm in S0 did not exhibit any self-healing after 48 h, indicating that the cementitious material alone was insufficient to heal surface cracks. However, surface cracks with initial widths of 0.26 mm, 0.39 mm and 0.39 mm in S1 (Figure 15c,d), S2 (Figure 15e,f) and S3 (Figure 15e,f), respectively, were able to completely heal within 48 h. This suggests that the microcapsules possess the capability to self-heal surface cracks in mortar. Notably, M3 demonstrated the most effective self-healing effect on surface cracks in the mortar, as it exhibited the largest healing crack width.

#### 3.6.2. Healing Rate

**Figure 16:**
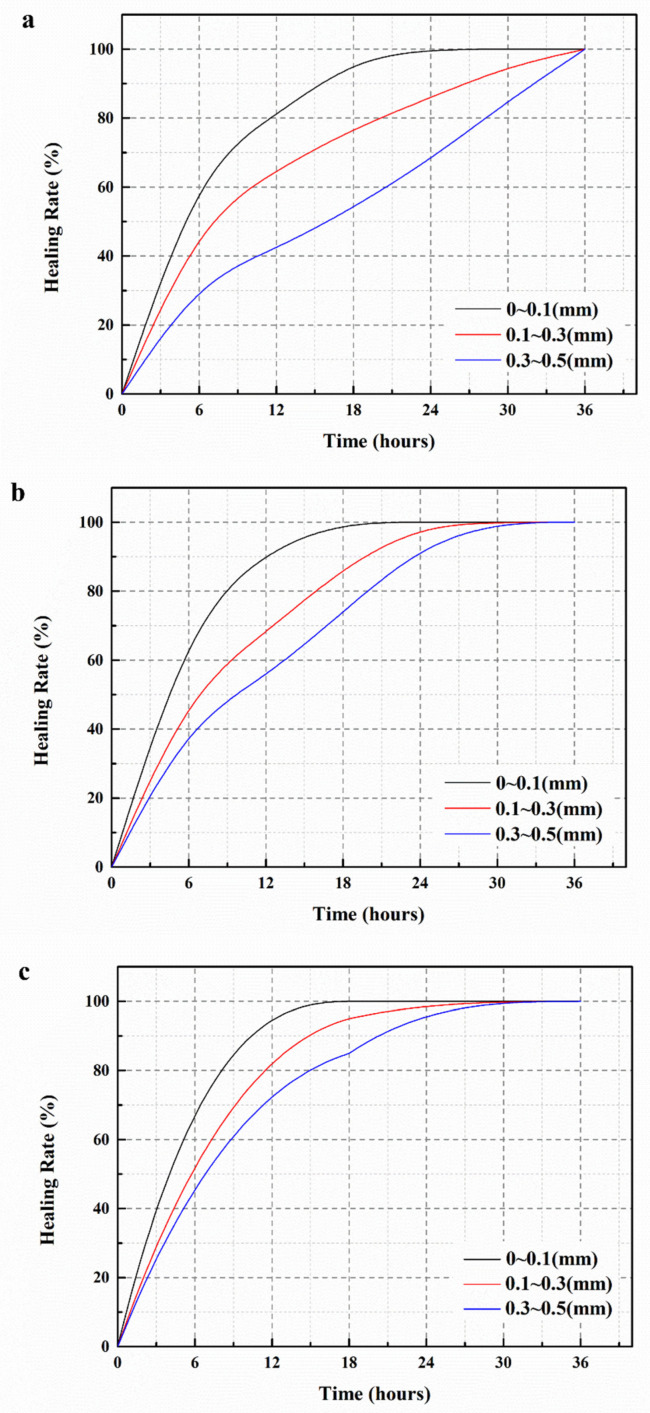
Surface cracks healing rates of mortars. (**a**) S1, (**b**) S2, (**c**) S3.

The healing rates of surface cracks for S1, S2 and S3 are presented in Figure 16. From Figure 16a, it can be observed that surface cracks in S1 with widths less than 0.1 mm can completely self-heal within 24 h, while cracks with widths ranging from 0.1 to 0.3 mm and 0.3 to 0.5 mm require 36 h for complete healing. Similarly, in Figure 16b, surface cracks in S2 with widths less than 0.1 mm only need 18 h to self-heal, and cracks with widths ranging from 0.1 to 0.3 mm and 0.3 to 0.5 mm can completely self-heal within 30 h. These results can be attributed to the splitting of the mortar, which creates the cracks. The stress exerted on the cracks damages the shells of M1 and M2, allowing the initially encapsulated IPDI in microcapsules to flow out and react with moisture, resulting in the formation of polyurea that fills the cracks. Figure 16c shows that surface cracks in S3 with widths less than 0.1 mm heal completely within 12 h, while cracks with widths ranging from 0.1 to 0.3 mm and 0.3 to 0.5 mm self-heal within 24 h. Comparing Figure 15a–c, it is evident that S3 requires significantly less time for self-healing and exhibits higher healing efficiency when the initial width of the surface cracks is the same. This improvement is attributed to the presence of ferrous powder in the shell material of M3, which generates heat when exposed to an external electromagnetic field, resulting in the timely breakage of the M3 microcapsules dispersed in S3 [39]. Additionally, the generated heat increases the temperature in the mortar, accelerating the reaction rate between IPDI and moisture and enhancing the self-healing rate of the cracks.

## 4. Conclusions

This study focuses on the development and characterization of three types of microcapsules, namely M1, M2 and M3, which utilize IPDI as the healing agent and employ polyethylene wax, polyethylene wax/nano-CaCO_3_ or polyethylene wax/ferrous powder as shell materials, respectively. The influence of these microcapsules on the mechanical properties, pore structure and permeability of mortars was thoroughly investigated by incorporating them into the mortar matrix. To assess the self-healing capability of the microcapsules, specimens (S0, S1, S2 and S3) underwent various treatments such as preload or splitting and were exposed to different environmental conditions, including room temperature or an applied magnetic field. The kinetics of the curing reaction between IPDI and moisture during the self-healing process were studied using quasi-first-order and quasi-second-order reaction kinetic models.

The results demonstrate that M1 had a significant positive effect on the mechanical properties, pore structure and permeability of the mortar, whereas M2 and M3 showed minimal impact on these properties. All three types of microcapsules, M1, M2 and M3, effectively improved the compressive strength retention, recovery of chloride diffusion coefficient, surface crack healing rate and maximum amplitude of the mortar. Furthermore, they contributed to the reduction of pores larger than 0.1 μm. Among the specimens, the self-healing ability for both internal damage and surface cracks followed the order of S1 < S2 < S3. Notably, specimen S3 exhibited remarkable improvements after self-healing, with a compressive strength retention of 92.2%, a percentage reduction of pores larger than 0.1 μm by 42.6%, a recovery of chloride diffusion coefficient by 78.9% and a maximum amplitude of 28.87 mV. Moreover, surface cracks with an initial width ranging from 0.3 to 0.5 mm were completely healed within a 24-h period. The self-healing process, involving the reaction between IPDI and moisture, was found to conform to a quasi-second-order reaction kinetic model.

By reducing the usage of ordinary Portland cement and incorporating more environmentally friendly supplementary cementitious materials (SCMs), carbon emissions and resource consumption can be effectively reduced. The application of electromagnetic inductive microcapsules in LC3 cementitious materials contributes to achieving sustainability goals. Future research can further optimize the mixture proportions and explore the combination of different environmentally friendly SCMs to enhance the sustainability and environmental friendliness of LC3 cement. Additionally, emphasis can be placed on assessing the long-term performance of LC3 cementitious materials with electromagnetic inductive microcapsules, including freeze-thaw resistance, chemical erosion resistance and durability. These investigations will provide essential guidance and evidence for practical engineering applications.

## Figures and Tables

**Table 1:** Oxide chemical compositions measured with XRF spectroscopy in weight %.

	OPC	Limestone	Calcined Clay	Gypsum
SiO_2_	22.97	0.75	64.08	14.05
Fe_2_O_3_	2.99	0.09	6.46	2.02
CaO	61.71	57.63	0.25	38.91
Al_2_O_3_	5.96	0.19	24.14	5.98
MgO	2.56	0.55	0.46	4.11
K_2_O	1.01	-	3.17	-
SO_3_	2.21	0.08	-	35.74
Na_2_O	0.04	-	-	-
TiO_2_	0.28	-	0.69	-
Loss on ignition	0.91	38.98	0.71	0.18

**Table 2:** Various indicators of microcapsules.

	M1	M2	M3
**Average Particle Size (μm)**	75	155	210
**Core Content (%)**	68.2	79.4	81.9
**Elastic Modulus (GPa)**	0.89	2.11	2.24
**Hardness (MPa)**	7.77	73.12	74.67
**Weight Loss Rate in 60 Days (%)**	7.98	1.55	1.43

**Table 3:** Mixture ratio of mortars.

Specimen	OPC	Calcined Clay	Limestone	Gypsum	Sand	Water	Microcapsules
**S0**	50	30	15	5	300	50	0
**S1**	50	30	15	5	300	50	4(M1)
**S2**	50	30	15	5	300	50	4(M2)
**S3**	50	30	15	5	300	50	4(M3)

**Table 4:** Quasi-first-order and quasi-second-order curing reaction kinetics data.

Specimen	Quasi-First-Order Curing Kinetics		Quasi-Second-Order Curing Kinetics	
	K_1_	R^2^	K_2_	R^2^
**S1**	0.919	0.479	1.506	0.995
**S2**	1.058	0.456	1.883	0.995
**S3**	1.959	0.618	6.098	0.999

## Data Availability

Not applicable.

## Supplementary Materials

The following supporting information can be downloaded at: https://www.mdpi.com/article/10.3390/polym15143081/s1, 1. Fabrication of Microcapsules; 2. Average Particle Size of Microcapsules; 3. Core Content of the Microcapsules; 4. Elastic Modulus and Hardness of Microcapsules; 5. Weight Loss Rate in 60 days of Microcapsules; 6. SEM; 7. Pore Size Distribution; 8. Ultrasonic Testing.
