# Supplementary material for: Influence of Electromagnetic Inductive Microcapsules on Self-Healing Ability of Limestone Calcined Clay Cement (LC3) Mortar
Source: Polymers (Basel). 2023 Jul 18;15(14):3081. doi: 10.3390/polym15143081 (PMC10384683; doi:10.3390/polym15143081)
Supplement: Supplementary file 1 [file polymers-15-03081-s001.zip › polymers-2466901-supplementary.pdf]

1. Fabrication of Microcapsules

Microcapsule 1 (M1):

A three-neck flask was used to melt 40 g of polyethylene wax in an oil bath at 125°C. Subsequently, 60 g of IPDI was added to the flask, and the resulting mixture was agitated for 150 minutes at 900 rpm. To induce a sharp drop in temperature, 400 mL of perfluoro-tributylamine was added to the flask, resulting in the formation of microcapsules (polyethylene wax-coated IPDI). The microcapsule suspension was then subjected to 40 min of ultrasound oscillation before being filtered.

Microcapsule 2 (M2):

A three-neck flask was used to melt 35 g of polyethylene wax in an oil bath at 125°C. Subsequently, 5 g of nano-$CaCO_3$ and 60 g of IPDI was added to the flask, and the resulting mixture was agitated for 150 minutes at 900 rpm. To induce a sharp drop in temperature, 400 mL of perfluoro-tributylamine was added to the flask, resulting in the formation of microcapsules. The microcapsule suspension was then subjected to 40 min of ultrasound oscillation before being filtered.

Microcapsule 3 (M3):

A three-neck flask was used to melt 35 g of polyethylene wax in an oil bath at 125°C. Subsequently, 5 g of ferrous powder and 60 g of IPDI was added to the flask, and the resulting mixture was agitated for 150 minutes at 900 rpm. To induce a sharp drop in temperature, 400 mL of perfluoro-tributylamine was added to the flask, resulting in the formation of microcapsules. The microcapsule suspension was then subjected to 40 min of ultrasound oscillation before being filtered.

2. Average Particle Size of Microcapsules

The average particle size was determined by a laser particle size analyzer (Mastersizer 2000, Malvern Instruments Ltd., Malvern, England). Before the test, microcapsules were placed in a drying box at 40 °C for 24 h. Then, 1 g microcapsules were dispersed by 50 mL deionized water in the analysis box. During the laser

diffraction measurement, particles were passed through a focused laser beam. These particles scatter light at an angle that was inversely proportional to their sizes. The angular intensity of the scattered light was measured via a series of photosensitive detectors. The size distribution of the after-sieving microcapsules was determined from the OM images of microcapsules using a commercial dimensional measurement software.

3. Core Content of the Microcapsules

A certain mass of microcapsules was first weighed and then thoroughly ground so that all the IPDI flowed out. Then, the residual shell was soaked in acetone for 24 hours. Finally, the residual shell was dried and weighed.

4. Elastic Modulus and Hardness of Microcapsules

The elastic modulus and hardness of microcapsules were tested by nanoindentation testing: First, the embedding agent (cold mount resin) was poured into a mold of Φ2.5 cm, and then microcapsules were added to it. After the embedding agent hardened, the surface of the microcapsules embedded in the cold mount resin was polished into a smooth sphere. Nanoindentation tests were performed by a Triboindenter (TI-900, Hysitron, USA) and fitted with a Berkovich tip. The indenter was in contact with the sample surface and had a calibrated trapezoidal load function defined as a loading rate of 20.00 mN/min, a holding time of 5 s at a maximum load of 10 mN, and an unloading rate of 20.00 mN/min. These mechanical values were recorded and plotted on a graph to create a load-displacement curve.

5. Weight Loss Rate in 60 days of Microcapsules

The weight loss rate in 60 days of microcapsules: the prepared microcapsules were selected and weighed directly. Then, the weighed microcapsules were stored in a curing cabinet (25°C, 50%RH). The weight loss of microcapsules was monitored after 60 days. Subtract the remaining weight from the initial weight, and then divide it by the initial weight.

6. SEM

The morphology of the microcapsules was observed by a scanning electron microscopy (SEM) (S-4800, Hitachi, Japan). Before the SEM observation, a small number of microcapsules were incised with a sharp blade and the IPDI inside the ruptured microcapsules was washed off with perfluoro-tributylamine in order to observe the shell thickness. The intact microcapsules and ruptured microcapsules were coated with a thin Pt layer on the surface. The intensity of the applied voltage is 3 kV.

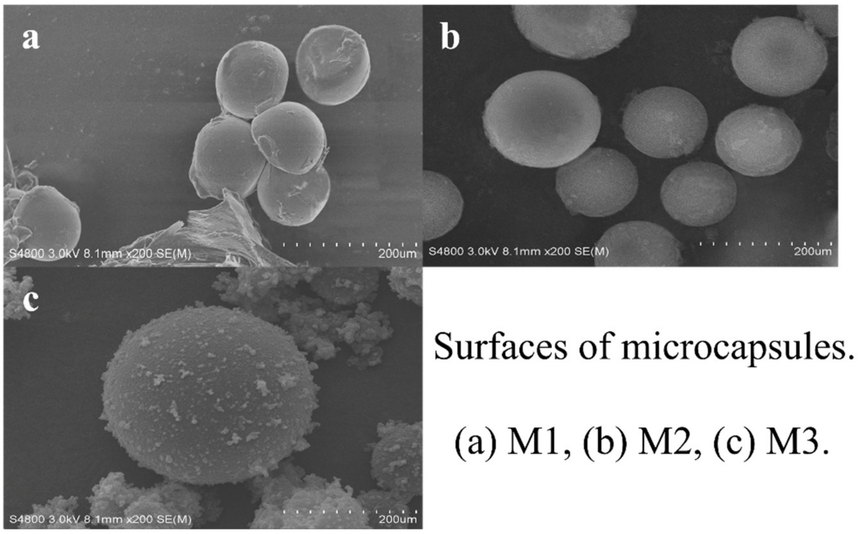

Figure S1. SEM image of microcapsules.

7. Pore Size Distribution

The pore size distribution of the mortar was investigated using a nuclear magnetic resonance spectrometer (MesoMR23, Suzhou Newman Analytical Instrument Co., Ltd, China) based on the relationship between the relaxation time of the hydrogen spectrum and the pore size of water molecules in the specimen. In this experiment, the pore size distribution of mortars subjected to pre-damage (60%f_a) and self-healing (25 °C, 50%RH) after 14 days was measured. Before testing, the specimens were wiped clean and saturated in a water vacuum for 24 h. The resonant frequency was 23.04 MHz, the magnet temperature was 32.00±0.02°C, and the probe diameter was 25 mm. The pore diameter was computed by formula (1).

$$\frac{1}{T_2}=\rho\left(\frac{S}{V}\right)_{pore} \tag{1}$$

where T_2 is the relaxation time of water in the pore (ms), ρ is the surface relaxation rate (70 μm/ms), and $(S/V)_{pore}$ is the pore surface area to volume ratio.

8. Ultrasonic Testing

A generator (AFG3022C, Tektronix Co., Ltd., China) was used to generate and transmit ultrasonic waves. An oscilloscope (MDO 3024, Tektronix Co., Ltd., China) was applied to receive and characterize the ultrasonic waves. The ultrasonic frequency was 107kHz and the voltage was controlled at ±5V. The radial piezoelectric ultrasonic transducer emitted ultrasonic waves when collecting data. The standard transducers were mounted on both sides of the specimen and the coupling agent was petroleum jelly. During the test, data and images were automatically connected to a computer. Ultrasound can propagate in different media with highly repeatable waveforms and frequencies when propagating at a steady state of energy. The mortars were tested before and after 3 days of self-healing.
